# Blastomere aggregation using phytohemagglutinin-L improves the establishment efficiency of porcine parthenogenesis-derived embryonic stem-like cell lines

**DOI:** 10.3389/fcell.2022.948778

**Published:** 2022-09-08

**Authors:** Joohyeong Lee, Lian Cai, Mirae Kim, Hyerin Choi, Dongjin Oh, Ali Jawad, Eunsong Lee, Sang-Hwan Hyun

**Affiliations:** ^1^ Veterinary Medical Center and College of Veterinary Medicine, Laboratory of Veterinary Embryology and Biotechnology (VETEMBIO), Chungbuk National University, Cheongju, South Korea; ^2^ Institute of Stem Cell & Regenerative Medicine (ISCRM), Chungbuk National University, Cheongju, South Korea; ^3^ Graduate School of Veterinary Biosecurity and Protection, Chungbuk National University, Cheongju, South Korea; ^4^ College of Veterinary Medicine, Kangwon National University, Chuncheon, South Korea

**Keywords:** blastomere aggregation, phytohemagglutinin-L, embryonic stem-like cells, parthenogenesis, pig

## Abstract

Aggregation of blastomeres is a promising method to improve the developmental competence of blastocysts and may be useful for the production of chimeric animals and the establishment of embryonic stem cell lines by increasing inner cell masses. Here, we determined the optimal conditions for blastomere aggregation using phytohemagglutinin-L (PHA-L) and examined PHA-L efficiency by comparing it with Well of the Well (WOW), a general blastomere aggregation method. As a result, we confirmed that treatment with 15 μg/ml PHA-L for 144 h was effective for blastomere aggregation and embryonic development of three zona-free 2-cell stage embryos (TZ2Es) after parthenogenetic activation (PA). The TZ2Es cultured with PHA-L showed a significantly (*p* < 0.05) higher blastomere aggregation rate than the WOW method (93.5 ± 1.9% vs. 78.0 ± 8.5%). In addition, our results demonstrated that TZ2Es aggregation through PHA-L improved the quality of PA-derived blastocysts and improved porcine embryonic stem-like cell (pESLCs) seeding efficiency and quality of colonies. It was also observed that PHA-L-derived pESLC could remain undifferentiated and exhibit typical embryonic stem cell pluripotency markers, embryoid body (EB)-forming ability, and differentiation into cell lineages of three germ layers. Pig blastomere aggregation technology is expected to improve embryo quality and the efficiency of embryonic stem cell establishment and embryoid-body formation. It can also be used in blastocyst complementation systems and in the production of chimeric animals.

## 1 Introduction

Porcine embryonic stem cells (ESCs) have been considered an important candidate for preclinical research on human disease models. In order to stably produce ESCs, high-quality blastocyst production must be supported. However, the quality of porcine embryos produced *in vitro* is lower than that produced *in vivo*, which is a major factor in reducing the establishment efficiency of porcine embryonic stem cell lines (Cheong et al., 2015). Production of high-quality blastocysts via aggregation can be a method to improve ESC efficiency and blastocyst developmental competence and may be useful for the production of chimeric animals and embryonic stem cell lines (He et al., 2013; Buemo et al., 2016; Ji et al., 2017). When the embryos formed through various assisted reproduction techniques are aggregated, some of these blastomeres can contribute to the formation of normally organized blastocysts. High-quality blastocysts produced through aggregation have been used to produce litter or ESCs. In the past several studies, a well of the well (WOW) system has been used for the aggregation of blastomeres (Hiriart et al., 2013; Saadeldin et al., 2015). A commercial culture dish with microwells can be used for blastomere aggregation using the WOW system. However, as this is not a specialized product for blastomere aggregation, it has the disadvantage of cost-inefficiency. For this reason, most laboratories use a pointed needle to fabricate and use artificial microwells (Tagawa et al., 2008; Dai et al., 2012; Rodriguez et al., 2021). This method removes the coating from the surface of the culture dish, which may cause the blastomeres to adhere to the base, and there is also a risk of damage to the embryo due to the rough surface. In this study, phytohemagglutinin-L (PHA-L) was used to examine the blastomere aggregation efficiency of porcine parthenogenetic-activated (PA) oocytes. PHA-L, a glycoprotein that binds to the cell surface via a specific glycol conjugate (Mallikarjunan et al., 2014), is a lectin extract from red kidney beans and consists only of the L-type subunit (isolectin L4, “leuco-agglutinin”). PHA-L has mitotic and strong cell aggregation activity. It binds to the T cell membrane and stimulates cell division and metabolic activity. PHA-L also can stimulate close contact between cell membranes. PHA-L has previously been used to successfully facilitate blastomere aggregation in mouse chimeras (Mintz et al., 1973), aggregation of SCNT-derived bovine embryos (Akagi et al., 2011), and optimize the SCNT procedure (Vajta et al., 2001; Oback et al., 2003; Du et al., 2006; Machebe et al., 2019). However, there have been no studies on blastomere aggregation using PHA-L in pigs. Therefore, in this study, we investigated whether PHA-L could improve the quality of blastocysts and the establishment efficiency of porcine embryonic stem-like cells (pESLCs) through blastomere aggregation of porcine PA embryos.

## 2 Materials and methods

### 2.1 Culture media

Unless otherwise stated, all chemicals were purchased from Sigma-Aldrich Corporation (St. Louis, MO, United States). Oocytes were allowed to mature in Medium-199 (M‐199; Invitrogen, Grand Island, NY, United States) supplemented with 0.91 mM pyruvate, 0.6 mM cysteine, 10 ng/ml EGF, and 1 μg/ml insulin, with 0.1% (w/v) polyvinyl alcohol (PVA) or 10% (v/v) porcine follicular fluid (PFF) as the base *in vitro* maturation (IVM) medium. Porcine zygote medium (PZM)‐3 containing 0.3% (w/v) bovine serum albumin (BSA) was used for *in vitro* culture (IVC) medium. On day 4, we transferred the embryos to PZM-3 drops containing 10% (v/v) FBS (ThermoFisher Scientific Waltham, MA, United States).

### 2.2 Oocyte collection and *in vitro* maturation

The ovaries of prepubertal gilts obtained from a local abattoir were transported to the laboratory at 34–37°C. Cumulus oocyte complexes (COCs) were aspirated from follicles of 3–8 mm in diameter. COCs with multiple layers of compacted cumulus cells were selected and washed three times in HEPES-buffered Tyrode’s medium (TLH) containing 0.05% (w/v) PVA (TLH‐PVA). COCs were placed in each well of a four-well multi-dish (Nunc, Roskilde, Denmark). Each well contained 500 µL of IVM medium with 10 IU/ml equine chronic gonadotropin and 10 IU/ml human chorionic gonadotropin (Intervet, Boxmeer, Netherland). After 22 h, the COCs were transferred to a fresh IVM medium without eCG/hCG and incubated for 22 h. IVM was performed at 39°C and 5% CO_2_ in a humid incubator (Astec, Fukuoka, Japan). After IVM, COCs were denuded by gentle pipetting with 0.1% hyaluronidase.

### 2.3 Parthenogenetic activation and embryo culture

After IVM, the MII-stage oocytes were selected to conduct PA as described by Lee et al. (Lee et al., 2021). The oocytes were washed twice in the activation solution containing 280 mM mannitol, 0.01 mM CaCl_2,_ and 0.05 mM MgCl_2_. The activation chamber connecting electrodes was filled with 1 ml of the activation solution. The oocytes were placed in the chamber and then activated with two direct-current pulses of 120 V/mm for 60 µs. Electro-activated oocytes were cultured in IVC medium containing 5 μg/ml cytochalasin B for 4 h to inhibit the extrusion of the second polar body and maintain diploid embryos. The PA embryos were washed three times with fresh IVC medium, transferred into 30 μl droplets of IVC medium under mineral oil, and then cultured at 39°C in a humidified atmosphere of 5% CO_2_, 5% O_2_, and 90% N_2_ for 7 days. Cleavage and blastocyst formation were evaluated on days 1 and 7, respectively, with the day of PA designated as day 0.

### 2.4 Zona pellucida removal and blastomere aggregation

According to the experimental design, the 1-, 2-, and 4-cell stage PA embryos were collected 4, 24, and 48 h after electrical activation, respectively. The collected embryos were incubated with Tyrode’s solution (Gibco) for 2 min to remove the zona pellucida. The zona pellucida-free blastomeres were aggregated using the PHA-L or WOW method. The blastomeres were aggregated by culturing for 20 min or 144 h in an IVC medium with 15 μg/ml or 150 μg/ml PHA-L. For the WOW system, micro well dishes were prepared 1 day before embryo culture. Glass ball with a diameter of about 550–600 µm was heated with an alcohol lamp, pressed down on the bottom of a polystyrene dish (Falcon REF351007, Coning, AZ, United States), and the pressure was maintained until the glass balls were sufficiently cooled (about 5 s). About 30 micro drops were prepared and used in a 60 mm diameter dish.

### 2.5 Experimental design

In Experiment 1, the effect of PHA-L treatment on the aggregation of blastomeres and embryonic development were investigated. In Experiment 2, the effect of the embryonic stage on blastomere aggregation and embryonic development was investigated. In Experiment 3, the effect of PHA-L concentration and treatment duration on blastomere aggregation and embryonic development was investigated. In Experiment 4, the aggregation efficiency of PHA-L or the WOW system and embryonic development were investigated. In Experiment 5, the ratio of inner cell mass (ICM) and the trophectoderm (TE) and cell death of blastocysts upon PHA-L treatment were investigated. In Experiment 6, the efficiency of establishing pESLCs using blastocysts derived from aggregated blastomeres was investigated. In Experiment 7, the characteristics of established pESLCs were analyzed.

### 2.6 Evaluation of developmental competence and total cell count

Day 0 was regarded as the day on which PA was initiated. On day 1 after PA, cleavage formation was analyzed. Blastocyst formation was assessed 7 days after PA. Aggregation efficiency refers to the proportion of aggregates that are still present in the form of a single lump on day 7 to the total cultured aggregates. To calculate the total cell number of blastocysts on day 7, the blastocysts were washed in TLH-PVA and fixed in 4% paraformaldehyde in PBS-PVA and stained for 5 min with 5 μg/ml of Hoechst-33342. Next, the blastocysts from each group were transferred to a drop of 100% glycerol on glass slides and gently covered with a coverslip. The stained blastocysts were observed using a fluorescence microscope (Nikon, Tokyo, Japan) at ×200 magnification.

### 2.7 TUNEL assay

To analyze blastocyst apoptosis, blastocysts were fixed with 4% (v/v) paraformaldehyde for 1 h at room temperature, washed with DPBS-PVA, and permeabilized with 0.1% (v/v) Triton X-100 in 0.1% (w/v) sodium citrate for 1 h at room temperature. After rinsing with DPBS-PVA, the embryos were stained with 45 ml of TUNEL-Label solution (Roche, Mannheim, Germany) supplemented with 5 ml TUNEL-Enzyme solution (Roche) for 1 h at 39°C in a dark, humidified atmosphere. Subsequently, nuclei were stained with 5 μg/ml Hoechst-33342 for 10 min blastocyst nuclei were analyzed by the method previously described under epifluorescence microscope (Kidson et al., 2004).

### 2.8 Establishment of embryonic stem-like cells

Preparation of mouse feeder cells was performed as described in Hwang et al. (Hwang et al., 2021). Mouse embryonic fibroblasts (MEFs) used as feeder cell layers were prepared from ICR mouse strains. The fetuses from ICR mice were sacrificed at 13.5 days of pregnancy. The fetal head, intestines, and legs were physically removed, and the remaining fetal tissues were physically crushed in the presence of DPBS (WELGENE, Inc., Daegu, Korea), followed by washing via centrifuging twice at 300 ×g for 2 min. The MEF culture medium was composed of Dulbecco’s modified Eagle medium (DMEM) containing 10% FBS, 1% non-essential amino acids, 1% glutamine, 0.1 mM β-mercaptoethanol, and 1% antibiotics-antimycotics (all from Gibco). MEFs were cultured at 37°C in a 5% CO_2_ humid incubator (Astec). Mitomycin C (10 μg/ml; Roche, Basel, Switzerland) was added to the MEF for 2–2.5 h to produce mitotically inactive ICR MEF feeder cells. These feeder cells were then plated at a density of 5 × 10^5^ cells/mL in a 4- or 6-well dish coated with 0.5% gelatin (WELGENE) containing the MEF culture medium. The ICR MEF feeder cells were typically plated 1–2 days before seeding the porcine blastocysts or pESLCs.

### 2.9 Measurement of blastocyst diameter and colony count of embryonic stem-like cells

Blastocysts or colonies of embryonic stem-like cells in each group were recorded at ×200 magnification using a digital camera (DS-L3; Nikon) attached to an inverted microscope (TE-300; Nikon). The diameter of the blastocyst or colony count of embryonic stem-like cells were measured using an image analysis software (ImageJ software, version 1.46; National Institutes of Health, Bethesda, MD, United States).

### 2.10 Culture of embryonic stem-like cells

On day 7 after PA, the blastocysts were seeded onto ICR MEFs at 5 × 10^5^ cells/ml. The pESLC culture medium was supplemented with 15% FBS and 4 ng/ml of basic fibroblast growth factor (bFGF; BioBud, Seoul, Korea) in low-glucose DMEM (Gibco). The pESLC medium was changed every day and subculture was performed every week. For subculture, only pESLC colonies were detached from the ICR MEF feeder cells, mechanically cut into several pieces, and placed on top of the new ICR MEF feeder cells.

### 2.11 Alkaline phosphatase activity detection

Alkaline phosphatase (AP) activity detection was performed as described in Hwang et al. (Hwang et al., 2021). pESLCs were washed three times with DPBS and fixed in 4% paraformaldehyde for 10 min at 25°C. Tris solution (0.1 M Tris, NaCl; pH 9.48) and NBT/BCIP chromogen solution (Roche) were added to the wells containing pESLCs and incubated for 20–60 min.

### 2.12 Gene expression analysis via quantitative real-time polymerase chain reaction (qRT-PCR)

First, the expression levels of pluripotency-related genes (*POU5F1*, *SOX2*, and *NANOG*) in the pESLCs from each treatment group were evaluated. Embryonic stem cells derived from each treatment group were sampled while maintaining three or more passages. The pESLC colonies isolated from the feeder cells were washed once in DPBS, centrifuged, and the supernatant was removed and stored at -80°C until analysis. Gene expression was analyzed by real-time PCR (CFX96 Touch Deep Well Real-Time PCR Detection System; BIO-RAD, Hercules, CA, United States). After mRNA extraction and cDNA synthesis, qRT-PCR was performed using 2 μl of cDNA template with 10 μl of 2X SYBR Premix Ex Taq (Takara Bio Inc., Shiga, Japan) containing primers specific to *POU5F1*, *NANOC*, and *SOX2* (Supplementary Table S1). Reactions were performed for 40 cycles under the following conditions: denaturation at 95°C for 30 s, annealing at 57°C for 15 s, and extension at 72°C for 30 s. Gene expression was quantified relative to the reference gene RN18S. Relative quantification was based on a comparison of the threshold cycle (Ct) at a constant fluorescence intensity. Relative mRNA expression was calculated using the following equation: R = 2^- [ΔCt sample−ΔCt control]^. Expression values were normalized to those of RN18S.

### 2.13 Immunofluorescence analysis

For immunofluorescence analysis of pESLC or EB, cells fixed with 4% paraformaldehyde were washed three times with PBS and permeabilized with 0.5% Triton X-100 for 30 min. Then, cells were coincubated with blocking solution (10% goat serum in PBS) and primary antibody overnight at 4°C. The primary antibodies used were anti-POU5F1 (AB19857; Abcam, Cambridge, United Kingdom, 1:200), anti-SOX2 (AB5603; Millipore, Temecula, CA, 1:200), anti-NANOG (AB70482; Abcam, 1:200), anti-cytokeratin 17 (AB49749; Abcam, 1:200), Desmin (MAB3430; Millipore, 1:200), anti-vimentin (MA5-11883; ThermoFisher, 1:100), anti-neurofilament (MAB1615; Millipore, 1:200), anti-desmin (MAB3430; Millipore, 1:200), and anti-cytokeratin 17 (AB49749; Abcam, 1:200). After washing three times with a washing medium (Tween20, Triton X-100, and PBS), the cells were incubated with the appropriate secondary antibodies. Secondary antibodies were applied, and the cells were incubated at room temperature (20–25°C) for 1 h. Nuclei were stained with Hoechst-33342. Stained cells were examined under a confocal microscope using the ZEN 3.5 Blue Edition software (Zeiss, Oberkochen, Germany).

### 2.14 Statistical analysis

Statistical analyses were performed using the Statistical Analysis System software (version 9.4; SAS Institute, Cary, NC, United States). The data were analyzed using a general linear model procedure followed by the least-significant-difference mean separation procedure when there were differences between treatments (*p* < 0.05). Percentage data were arcsine-transformed prior to analysis to maintain homogeneity of variance. The results were expressed as mean ± standard error of the mean (SEM).

## 3 Results

### 3.1 Effect of PHA-L on the aggregation efficiency and developmental competence of PA embryos

We evaluated the effect of PHA-L on the aggregation efficiency and developmental competence of the PA embryos. When 4-cell stage PA blastomeres were treated with PHA-L at 150 μg/ml for 20 min, the aggregation rate was significantly increased compared to the untreated group (57.9 ± 5.1% vs. 17.5 ± 11.8%). However, there was no significant difference in the blastocyst development rate (66.7–83.3%) and the number of cells (71.5–88.0 cells) (Table 1).

**TABLE 1:** Effects of Phytohemagglutinin (PHA-L) on the aggregation efficiency and developmental competence of parthenogenetic embryos in pigs.

Treatment		No. of embryo used in the examined^a^	% of aggregated embryo	% of embryos developed to blastocyst	No. of cells in blastocyst
PHA-L	Time				
-	-	66	17.5 ± 11.8^b^	66.7 ± 33.3	71.5 ± 16.5
150 μg/ml	20 min	72	57.9 ± 5.1^a^	83.3 ± 16.7	88.0 ± 6.8

a Four replicates.

^a,b^Different superscript letters indicate a significant difference within a column (*p* < 0.05).

Three denuded 4-cell stage embryos were aggregated in PZM-3, supplemented with 150 μg/ml PHA-L, for 20 min.

### 3.2 Effect of embryo stage on aggregation efficiency and developmental competence of PA embryos

We evaluated the effects of different embryonic stages (1-, 2-, and 4-cell) on the aggregation efficiency and developmental competence of PA embryos. When PA embryos of each cell stage were treated with 150 μg/ml of PHA-L for 20 min, the aggregation rate of 2-cell stage blastomeres was significantly higher than that of the 1- or 4-cell stage blastomeres (75.2 ± 9.1 vs. 43.6–57.5%). In addition, the number of cells in the blastocyst was significantly increased when aggregating 2-cell stage embryos (58.8 ± 4.8%) than when aggregating 1-cell stage embryos (71.3 ± 3.1%). There was no significant difference in blastocyst development rate according to cell stage. (Table 2).

**TABLE 2:** Effects of embryo stage on the aggregation efficiency and developmental competence of parthenogenetic embryos in pigs.

Treatment		No. of embryo used in the examined^a^	% of aggregated embryo	% of embryos developed to blastocyst	No. of cells in blastocyst
Stage of embryo	Time after activation (h)				
1-cell	4	93	43.6 ± 4.7^b^	95.0 ± 5.0	71.3 ± 3.1^b^
2-cell	24	99	75.2 ± 9.1^a^	100.0 ± 0.0	85.8 ± 4.8^a^
4-cell	48	90	57.5 ± 1.4^b^	86.7 ± 8.2	75.7 ± 3.2^a,b^

a Five replicates.

^a,b^Different superscript letters indicate a significant difference within a column (*p* < 0.05).

1-, 2- and 4-cell stage denuded embryos were aggregated in PZM-3, supplemented with 150 μg/ml PHA-L, for 20 min.

### 3.3 Effect of PHA-L concentration and treatment duration on blastomere aggregation and developmental competence of PA embryos

Three zona-free 2-cell stage embryos (TZ2Es) were aggregated in an IVC medium supplemented with 15 or 150 μg/ml of PHA-L for 20 min or 144 h. The highest rate of blastocyst development was observed following treatment with 15 μg/ml of PHA-L for 144 h, compared to other treatment groups (100% vs. 72.4–83.8%). However, the number of cells in the blastocyst and the aggregation rate of blastomeres were not affected by the treatment duration and concentration of PHA-L (Table 3).

**TABLE 3:** Effects of culture time with Phytohemagglutinin (PHA-L) and developmental competence of parthenogenetic embryos in pigs.

Treatment		No. of embryo used in the examined^a^	% of aggregated embryo	% of embryos developed to blastocyst	No. of cells in blastocyst
PHA-L	Time				
15 μg/ml	20 min	69	77.1 ± 10.4	72.4 ± 12.0^b^	86.7 ± 7.3
15 μg/ml	144 h	63	81.3 ± 7.1	100.0 ± 0.0^a^	84.7 ± 3.6
150 μg/ml	20 min	66	74.7 ± 9.2	80.4 ± 7.1^b^	83.7 ± 4.3
150 μg/ml	144 h	63	91.7 ± 4.8	83.8 ± 5.5^a,b^	82.3 ± 4.7

a Four replicates.

^a,b^Different superscript letters indicate a significant difference within a column (*p* < 0.05).

2cell stage denuded embryos were aggregated in PZM-3, supplemented with 15 or 150 μg/ml PHA-L, for 20 min or 144 h.

### 3.4 Effect of PHA-L on aggregation efficiency and developmental competence of PA embryos in pigs

**FIGURE 1:**
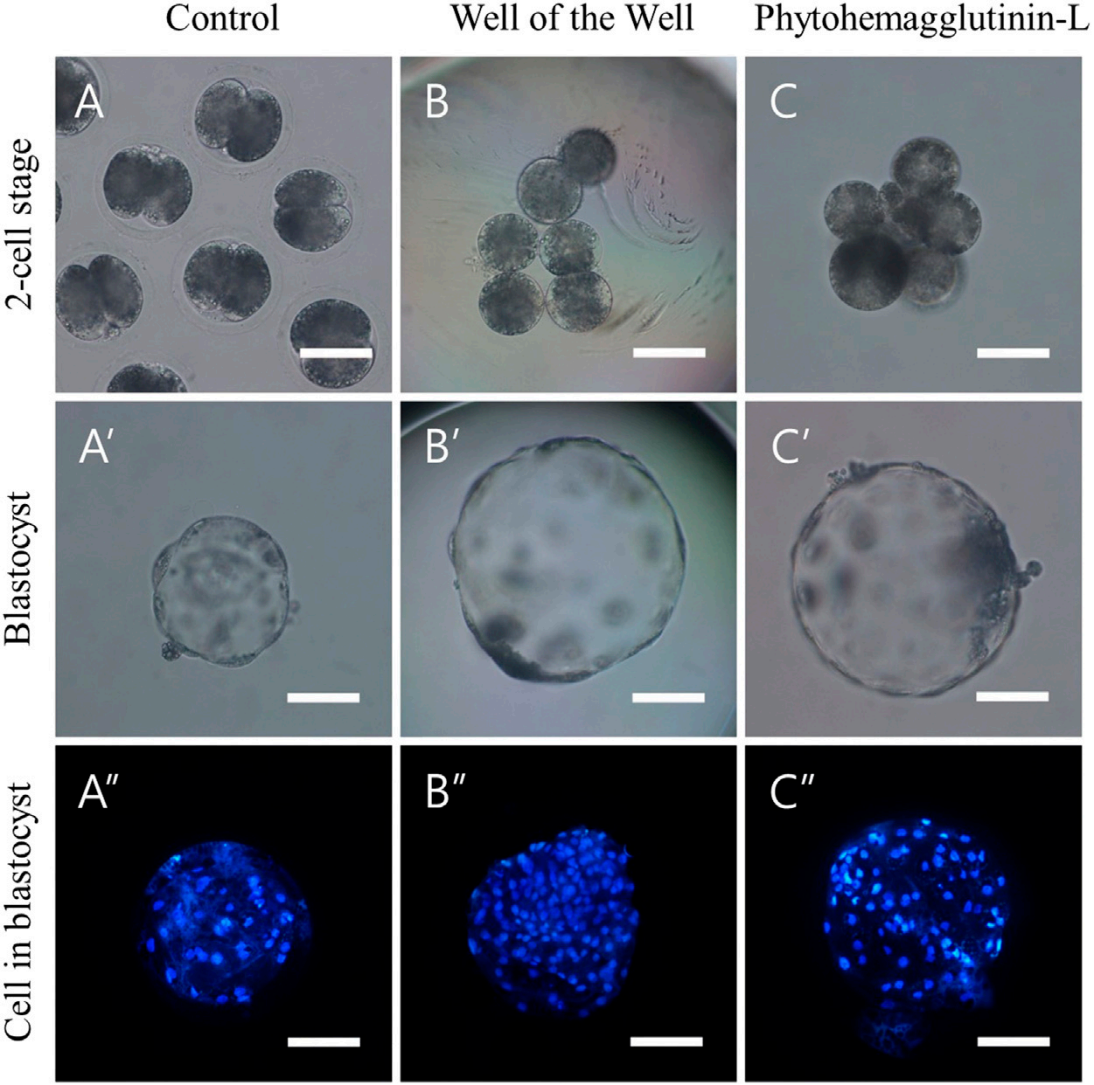
Aggregation of 2-cell stage blastomeres after parthenogenesis improves the quality of blastocysts. **(A)** Normal 2-cell embryos **(B)** 3x zona-free blastomeres aggregated by the well of the well (WOW), and **(C)** phytohemagglutinin-L (PHA-L) methods. Optical (A′–C′) and fluorescence (A″–C″) microscope images of blastocysts derived from normal, WOW, and PHA-L methods. Scale bar = 100 um.

We investigated the aggregation efficiency of PHA-L by setting the WOW method, commonly used for blastomere aggregation, as a positive control. TZ2Es were aggregated using the WOW or PHA-L methods to investigate the aggregation rate and blastocyst development rate. The PHA-L method showed a significantly higher blastomere aggregation rate than the WOW method (93.5 ± 1.9% vs. 78.0 ± 8.5%). However, there was no significant difference between the blastocyst development rate (85.2–90.4%) and the number of cells in the blastocyst (93.8–94.9 cells) (Figure 1; Table 4).

**TABLE 4:** Effects of Phytohemagglutinin (PHA-L) on the aggregation efficiency and developmental competence of parthenogenetic embryos in pigs.

Type of embryo	Methods of blastomere aggregation	No. of oocytes cultured (aggregate)	*% of aggregated embryo^a^	% of embryos developed to blastocyst		No. of cells in blastocyst
				/Embryos	/Aggregated embryos	
2-cell stage embryo	_	65	_	65.5 ± 4.9	_	39.7 ± 3.1^a^
TZ2Es^b^	WOW^c^	165 (55)	78.0 ± 8.5^a^	_	90.4 ± 1.0	93.8 ± 5.8^b^
TZ2Es^b^	PHA-L^d^	171 (57)	93.5 ± 1.9^b^	_	85.2 ± 4.0	94.9 ± 5.4^b^

^a,b^Different superscript letters indicate a significant difference within a column (*p* < 0.05).

^a^Four replicates.

b TZ2Es: Three zona-free 2-cell stage embryos.

c TZ2SEs, were aggregated with well of well system.

d TZ2Ses were aggregated in PZM-3, supplemented with 15 μg/ml PHA-L, for 144 h.

### 3.5 Effect of PHA-L on the TE and ICM numbers and blastocyst apoptosis

When TZ2Es were aggregated using the WOW and PHA-L methods, the ICM ratio of the blastocysts was significantly higher than that of the blastocysts derived from non-aggregated blastomeres (33.6–37.6 vs. 18.9%). However, the ICM and TE numbers and the cell death rate of blastocysts did not differ significantly between the two aggregation methods (Table 5).

**TABLE 5:** Effects of Phytohemagglutinin (PHA-L) on the number of inner cell mass (ICM) the trophectoderm (TE) and apoptosis of blastocyst in pigs.

Type of embryo	Methods of blastomere aggregation	No. of blastocysts evaluated	No. of			ICM/Total ratio	No. of blastocysts evaluated	% of TUNEL positive cell in blastocyst
			ICM cells	TE cells	Total cells			
2-cell stage embryo	_	27	7.4 ± 1.1^a^	30.2 ± 2.0^a^	37.6 ± 2.6^a^	18.9 ± 2.3^a^	27	4.4 ± 0.7
TZ2Es^a^	WOW^b^	30	31.1 ± 3.0^b^	63.1 ± 4.9^b^	94.2 ± 5.2^b^	33.6 ± 2.9^b^	21	3.9 ± 0.7
TZ2Es^a^	PHA-L^c^	31	32.3 ± 2.9^b^	52.7 ± 3.6^b^	85.1 ± 3.8^b^	37.6 ± 3.0^b^	29	3.0 ± 0.4

^a,b^Different superscript letters indicate a significant difference within a column (*p* < 0.01).

a TZ2Es: Three zona-free 2-cell stage embryos.

b TZ2Es were aggregated with well of well system.

c TZ2Es were aggregated in PZM-3, supplemented with 15 μg/ml PHA-L, for 144 h.

### 3.6 Effect of PHA-L on blastocyst diameter and outgrowth of colonies from the blastocyst of PA embryos

When TZ2Es were aggregated using WOW and PHA-L methods, blastocyst diameter was significantly higher than that of the blastocysts derived from non-aggregated blastomeres (260.3–266.5 vs.183.6 μm). Moreover, aggregated blastocysts significantly increased the rate of outgrowing colonies and colony diameter at 7 days post-seeding compared to non-aggregated blastocysts (43.6–44.3 vs. 29.2%, 658.2–660.7 vs. 432 μm, respectively) (Table 6).

**TABLE 6:** Effects of Phytohemagglutinin (PHA-L) on diameter of blastocyst and outgrowth of colonies from blastocyst of parthenogenetic embryos in pigs.

Type of embryo	Methods of blastomere aggregation	No. of blastocysts evaluated	Diameter of blastocyst (μm)	No. of blastocyst seeded	No. of outgrowing colonies at 7 days after seeding (%)	Diameter of a colonies at 7 days after seeding (μm)
2-cell stage embryo	_	20	183.6 ± 10.4^a^	52	14 (29.2)^a^	432.1 ± 52.7^a^
TZ2Es^a^	WOW^b^	20	266.5 ± 15.5^b^	69	28 (43.6)^b^	658.2 ± 78.2^b^
TZ2Es^a^	PHA-L^c^	20	260.3 ± 14.5^b^	66	29 (44.3)^b^	660.7 ± 54.0^b^

^a,b^Different superscript letters indicate a significant difference within a column (*p* < 0.05).

a TZ2Es: Three zona-free 2-cell stage embryos.

b TZ2Es were aggregated with well of well system.

c TZ2Es were aggregated in PZM-3, supplemented with 15 μg/ml PHA-L, for 144 h.

### 3.7 Characterization of ESLCs

**FIGURE 2:**
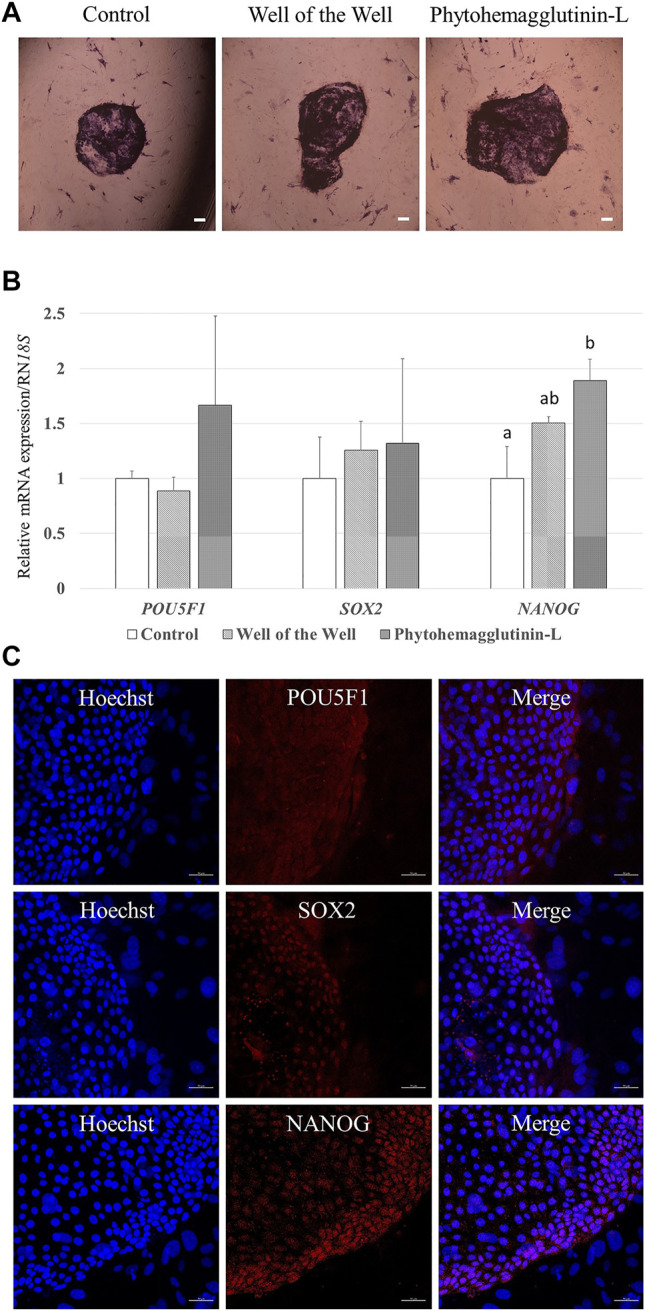
Characterization of porcine embryo stem-like cells (pESLCs) from each treatment group. **(A)** Alkaline phosphatase stain; alkaline phosphatase acts on a substrate to form a purple colored. Scale bar = 200 μm. **(B)** Expression of the pluripotency-related genes (*POU5F1*, *SOX2*, and *NANOG*) in pESLCs from each treatment. A total of four replicates were included. ^a, b^ Different superscript letters indicate significant differences within the columns (*p* < 0.05). **(C)** Expression of pluripotent markers (*POU5F1*, *SOX2*, and *NANOG*) in pESLCs from phytohemagglutinin-L, as assessed by immunofluorescence. Nuclei and pluripotent markers are shown in blue and red, respectively. Scale bar = 50 μm.

**FIGURE 3:**
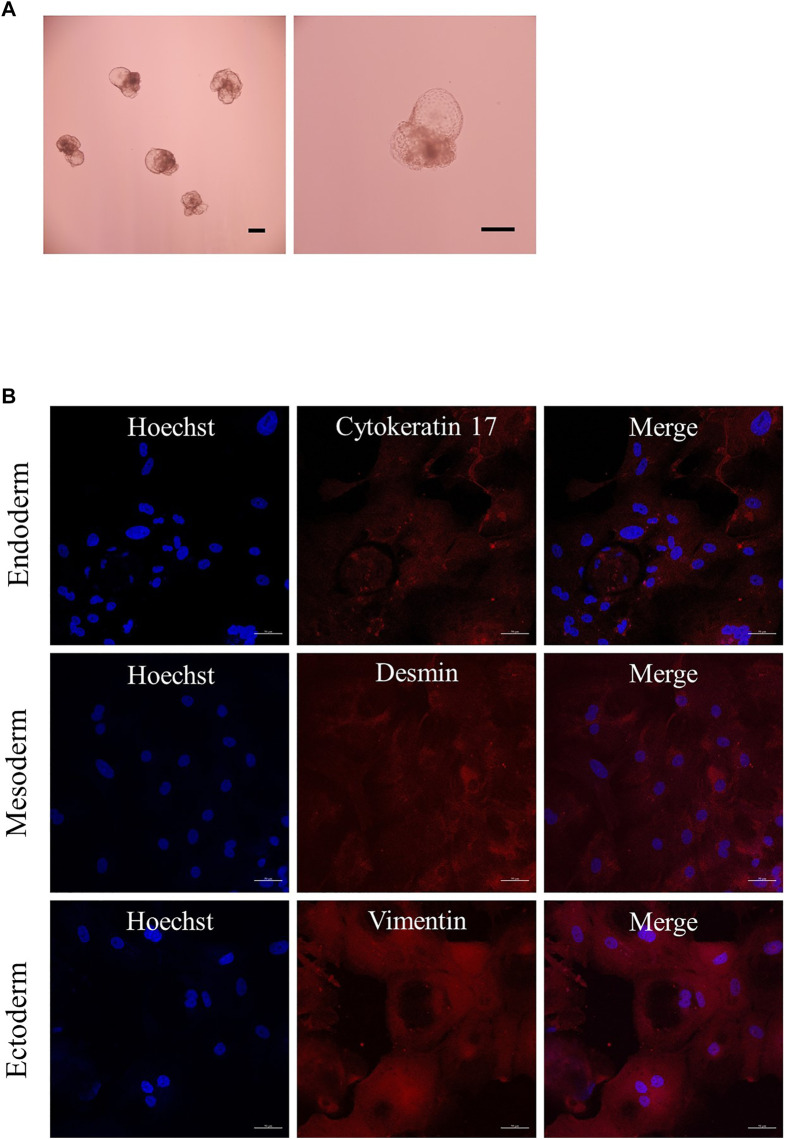
Expression of differentiation in embryoid bodies (EBs). **(A)** EBs derived from porcine embryo stem-like cells with PHA-L. Scale bar = 200 μm. **(B)** Expression of differentiation markers cytokeratin 17 (endoderm), desmin (mesoderm), and vimentin (ectoderm) from EBs by immunofluorescence analysis. Nuclei and differentiation markers are shown in blue and red, respectively. Scale bar = 50 μm.

The characteristics of the established porcine ESLC lines were analyzed. First, AP staining showed that pESLC lines in all experimental groups had AP activity in colonies except MEF feeder cells (Figure 2A). It was confirmed through qRT-PCR analysis that the undifferentiated pluripotent cell markers *POU5F1*, *SOX2*, and *NANOG* were expressed in the pESLC lines derived from all experimental groups (Figure 2B). In addition, the expression of the NANOG gene was significantly increased in the PHA-L treatment group compared to that of the control group. Expression of POU5F1, SOX2, and NANOG in pESLCs derived from embryos aggregated with PHA-L was confirmed through fluorescence immunostaining (Figure 2C). EBs were formed using pESLCs derived from blastomeres aggregated with PHA-L treatment (Figure 3A). We confirmed the normal differentiation of EBs into endoderm, mesoderm, and ectoderm by fluorescence immunostaining (Figure 3B).

## 4 Discussion

Already, embryo aggregation method is widely used in various mammals to improve production efficiency by compensating for developmental deficits *in vitro* embryos. In particular, pigs have been reported to respond positively to embryonic aggregation with increases in blastocyst formation rate, total cell number, ICM/TE ratio and cell viability. It has been reported that blastomere aggregation improves embryonic development by inducing a decrease in the level of apoptosis and an improvement in cell reprogramming (Lee et al., 2007; Terashita et al., 2011). In this study, the hemagglutinin PHA-L was used to induce the aggregation of porcine PA blastomeres. Porcine PA embryos cannot produce live offspring, but are a good material for embryo experiments. Compared to other artificial reproductive technologies, PA embryos are less affected by external factors (quality of donor cells or sperm) and have the advantage of a simple production method. In the last decade, blastomere aggregation using PHA-L was performed in some studies. However, there are no studies examining the effect of PHA-L in porcine blastomere. Previous studies reported that blastomere aggregation was induced using 150 μg/ml to 300 mg/ml PHA-L in cows (Simmet et al., 2015) and mice (Tarkowski et al., 2002), respectively. In our first experiment, we examined the effect of 150 μg/ml PHA-L treatment on the aggregation rate of blastomeres and the preimplantation embryonic development after PA. It was confirmed that PHA-L treatment significantly increased the aggregation rate of blastomeres compared to the untreated group and did not negatively affect blastocyst development rate and the number of cells in blastocysts. Next, an experiment was performed to investigate the optimal conditions for blastomere aggregation using PHA-L. First, to examine the effect of the embryo stage on blastomere aggregation, 1-, 2-, and 4-cell stage embryos were used. Embryos after the 4-cell stage are difficult to distinguish from embryos with fragmented blastomeres, so these were not used in the experiment. The 2-cell stage embryos collected 24 h after activation showed an aggregation efficiency significantly higher than that of the 4-cell stage embryos collected 48 h after activation, and embryonic development also showed higher efficiency than other embryonic cell cycles. In general, embryos at the 4- to 8-cell stage are used for blastomere aggregation in pigs (Ji et al., 2017), but in our results, high efficiency was observed when using blastomeres from embryos at an earlier stage than the 4-cell stage. This result may be related to the activation of the embryonic genome. The early embryonic genome is transcriptionally quiescent, and the first few cleavages are regulated by maternal factors stored in the oocyte until zygotic genome activation (Zhang et al., 2022). Porcine zygotic genome activation was confirmed to occur at the 4-cell stage (Cao et al., 2014). Abnormal zygotic genome activation can lead to embryonic developmental arrest which is an important cause of early embryonic loss in animals. Therefore, it may be more efficient to utilize 2-cell stage blastomeres that have not undergone zygote genome activation for blastomere aggregation. Based on our experimental results, only 2-cell stage embryos after activation stimulation were used for subsequent experiments. We investigated aggregation efficiency and embryonic development upon treatment with 15 or 150 μg/ml of PHA-L for 20 min or 144 h, respectively, using TZ2Es. We found no significant difference in the rate of blastomere aggregation between the two PHA-L concentration treatment groups. When investigating aggregation efficiency and embryonic development according to the PHA-L treatment time, the PHA-L treatment group (144 h) showed a significantly higher blastocyst development rate than the 20 min treatment group. These results indicate that continuous PHA-L treatment during the *in vitro* culture period strongly maintains the aggregation of blastomeres, leading to a high rate of blastocyst development. Furthermore, we indirectly confirmed that long-term PHA-L treatment did not induce toxicity by examining the ratio of the number of apoptotic cells in blastocysts. Based on the results of these experiments, we concluded that the highest embryonic development rate was obtained when the 2-cell stage embryos collected 24 h after electrical stimulation were treated with 15 μg/ml of PHA-L for 144 h. This condition was used in subsequent experiments. The WOW method is the most commonly used method for blastomere aggregation. It has been reported that the aggregation of blastomeres derived from somatic cell nuclear transfer using the WOW technique is an effective method for producing high-quality blastocysts in pigs (Buemo et al., 2016). In this study, we set a non-aggregated embryo as the negative control and the WOW method as the positive control to compare the efficiency of blastocyst aggregation through PHA-L. Pigs have a longer preimplantation period than mice and humans during early embryonic development. They also have an ICM that is considered a pluripotent progenitor population (Alberio and Perez, 2012). In the results of this study, the aggregated embryos showed a significantly higher blastocyst rate, the number of cells in the blastocyst, and ICM ratio, compared to the control. It can be inferred that the increase in the proportion of ICM of blastocysts derived from blastomere aggregation is not only proportional to the number of aggregated embryos but also increased as a result of the interaction between blastomeres. In addition, blastomere aggregation through PHA-L exhibited a significantly higher aggregation efficiency than the WOW technique. In the WOW method, some blastomeres separated from the main aggregate are often observed at the beginning of culture due to weak adhesion between blastomeres. In contrast, the blastomeres agglomerated using PHA-L had strong adhesion; thus, the aggregation efficiency was improved compared to the WOW method. However, there were no significant differences in the proportion of blastocysts, the number of cells in the blastocyst, ICM rate, and apoptosis rate in the embryos aggregated through each method. We examined whether PHA-L had a synergistic effect when treated with various types of culture dish surfaces (U-type and WOW with agarose plate) in Supplementary Table S2. However, no significant differences were observed in embryo aggregation and blastocyst development rates. In general, pluripotent stem cells of other species, especially farm animals, have not reached the full biological characteristics of murine embryonic stem cells. This is also true for pigs (Brevini et al., 2012; Xue et al., 2016). Despite numerous reports, no conclusive results have been obtained on the isolation and propagation of porcine embryonic stem cell lines, and the establishment of pluripotent cells from the pig has remained an elusive goal. To establish putative porcine embryonic stem cell lines, blastocyst quality is among the most crucial factors affecting outgrowth and colony formation (Siriboon et al., 2015). In this study, improved blastocyst quality by blastomere aggregation improved outgrowth and colony quality in establishing pESLCs. This result could be attributed to the high ICM number of blastocysts. This is consistent with previous studies already reported on pigs (Tan et al., 2012). We confirmed the pluripotency of pESLCs established from PHA-L-derived blastocysts through AP staining, the expression of pluripotency marker genes (*POU5F1*, *SOX2*, and *NANOG*), and fluorescence immunostaining. Pluripotent stem cells typically have higher levels of alkaline phosphatase activity than differentiated cell types. In our results, it can be seen that the ESC colonies show a higher purple color than the feeder cells (Figure 2A). These results confirm that the transplanted blastocysts have been converted into pluripotent stem cells. POU5F1 (also known as OCT4), SOX2 and NANOG are all transcription factors essential for maintaining the pluripotent embryonic stem cell phenotype (Rodda et al., 2005). These three pluripotency factors contribute to a complex molecular network that regulates a number of genes controlling pluripotency. In our results, it was confirmed that POU5F1, SOX2, and NANOG genes were expressed through immunostaining in pESLCs derived from PHA-L, unlike feeder cells (Figure 2C). Interestingly, pESLCs derived from PHA-L showed significantly higher *NANOG* expression than control (pESLCs from non-aggregated embryos). Although these results show that embryonic stem cells derived from blastomere aggregation are more pluripotent, further studies are needed to understand how blastomere aggregation through PHA-L increases the expression of *NANOG* in pESLCs. ICM-derived embryonic stem cells can proliferate indefinitely *in vitro* and can differentiate into cells of all three germ layers (Pan and Thomson, 2007). Finally, we demonstrated that PHA-L-derived pESLCs could contribute to the formation of normal pESLCs by confirming that they are capable of differentiation into endoderm, mesoderm and ectoderm after EB formation. In conclusion, TZ2E aggregation through PHA-L improved the quality of PA-derived blastocysts, as well as pESLC seeding efficiency and quality of colonies. It was also demonstrated that PHA-L-derived pESLCs could remain undifferentiated and exhibit typical ES cell pluripotency markers, EB-forming ability, and differentiation into cell lineages of three germ layers. This technique is expected to improve embryo quality and embryonic stem cell establishment efficiency and can be utilized for blastocyst complementation systems and the production of chimeric animals.

## Data Availability

The original contributions presented in the study are included in the article/Supplementary Material, further inquiries can be directed to the corresponding author.
