# Supplementary material for: Blastomere aggregation using phytohemagglutinin-L improves the establishment efficiency of porcine parthenogenesis-derived embryonic stem-like cell lines
Source: Front Cell Dev Biol. 2022 Sep 8;10:948778. doi: 10.3389/fcell.2022.948778 (PMC9493121; doi:10.3389/fcell.2022.948778)
Supplement: Supplementary file 1 [file Table1.DOCX]

| Supplementary Table 1. Primers used for gene expression analyses. | | | | | |
| --- | --- | --- | --- | --- | --- |
| Gene | Primer sequences (5′-3′) | | Product size (bp) | Tm | Gen Bank accession number |
| *RN18S* | F: | CGCGGTTCTATTTTGTTGGT | 219 | 56 | NR_046261.1 |
|  | R: | AGTCGGCATCGTTTATGGTC |  | 58 |  |
| *POU5F1* | F: | GCGGACAAGTATCGAGAACC | 200 | 58 | XM_021097869.1 |
|  | R: | CCTCAAAATCCTCTCGTTGC |  | 57 |  |
| *SOX2* | F: | CCGTGGTTACCTCTTCTTCC | 186 | 58 | NM_001123197.1 |
|  | R: | AGAGAGGCAGTGTACCGTTG |  | 59 |  |
| *NANOG* | F: | TAAAACCACTGCCCACATCT | 131 | 57 | XM_021092390.1 |
|  | R: | CTGCCTCTGAAATCTGTCGT |  | 58 |  |

| Supplementary Table 2. Effects of plate type on the aggregation efficiency and developmental competence of parthenogenetic embryos in pigs | | | | |
| --- | --- | --- | --- | --- |
| Type of plate | No. of embryo used in the examined * | % of aggregated embryo | % of embryos developed to blastocyst | No. of cells in blastocyst |
| Normal | 102 | 87.2 ± 2.4 ^a^ | 96.9 ± 3.1 ^a^ | 91.4 ± 4.7 |
| WOW | 87 | 84.6 ± 7.9 ^a^ | 92.2 ± 4.8 ^ab^ | 86.5 ± 5.3 |
| U-type | 87 | 78.3 ± 6.1 ^ab^ | 95.8 ± 4.2 ^a^ | 86.0 ± 7.1 |
| WOW with agarose plate | 87 | 58.5 ± 9.0 ^b^ | 66.7 ± 15.6 ^b^ | 77.0 ± 4.3 |
| * Four replicates.  ^a, b^ Different superscript letters indicate a signiﬁcant difference within a column (p < 0.05).  Three zona-free 2-cell stage embryos were aggregated with 15 ug/mL PHA-L for 144 h. | | | | |
